# Provider- and System-Level Barriers and Facilitators to Colonoscopy and Multi-Target Stool DNA for Colorectal Cancer Screening in Rural/Remote Alaska Native Communities

**DOI:** 10.3390/ijerph20227030

**Published:** 2023-11-07

**Authors:** Diana Redwood, Melissa Toffolon, Christie Flanagan, John Kisiel, Judith Salmon Kaur, Lauren Jeffries, Manusake Zenku, Jennifer Lent, Joseph Bachtold

**Affiliations:** 1 Alaska Native Tribal Health Consortium, 3900 Ambassador Dr., Anchorage, AK 99508, USA; 2 Actionable Data Consulting, P.O. Box 873427, Wasilla, AK 99687, USA; mt@actionabledataconsulting.com; 3 Mayo Clinic, 200 1st Street SW, Rochester, MN 55905, USA; 4 Yukon-Kuskokwim Health Corporation, 829 Chief Eddy Hoffman Hwy, Bethel, AK 99559, USA

**Keywords:** Alaska Native, cancer screening, colorectal cancer, health care, provider, screening barriers, colorectal neoplasms/prevention and control, early detection of cancer, health personnel, surveys and questionnaires

## Abstract

The Alaska Tribal Health System is working to increase colorectal cancer (CRC) screening among Alaska Native people, who experience the highest CRC rates in the world. This study examined CRC screening provider- and system-level barriers and facilitators from the perspective of healthcare providers serving Alaska Native people in rural/remote communities. A total of 28 provider (physicians, advanced practice, and Community Health Aides/Practitioners) interviews were held from 1 February to 30 November 2021. Colonoscopy provider-level barrier themes included time, competing priorities, and staffing, while system-level barriers included travel costs, weather, and the COVID-19 pandemic. Multi-target stool DNA (mt-sDNA) barrier themes included test viability and unfamiliarity, and previous stool tests experiences. For both tests, limited medical record reminders was a major barrier. Facilitator themes for both tests included community outreach, cultural competency and patient navigation, and clinic/system improvements. In-depth interviews with tribal health providers showed that adding mt-sDNA testing may help address system-level colonoscopy barriers such as waitlists and travel costs, but other barriers remain. Further research is needed into patient barriers and facilitators, as well as the effectiveness of integrating mt-sDNA into a geographically dispersed tribal health system to reduce cancer disparities and build equity in CRC prevention among Alaska Native people.

## 1. Introduction

Colorectal cancer (CRC) early detection and prevention through screening has been associated with a decline in CRC incidence and mortality across the United States (US), and is recommended for US adults ages 45–75 [1]. Alaska Native people have the highest CRC mortality in the world [2], over twice as high as US Whites (37.4 per 100,000 vs. 13.4 per 100,000 persons) [3]. This substantial cancer health disparity has led the Alaska Tribal Health System to focus efforts on CRC screening in this population. As a result, CRC screening rates are now comparable between Alaska Native and non-Alaska Native people in the state (71.1% vs. 69.6%, respectively), although rates vary by region [4,5]. Despite this success, CRC incidence and mortality among Alaska Native people have not declined over time as in other racial/ethnic groups [1,3,6]. There is a critical need to reduce the structural inequality related to CRC faced by Alaska Native communities.

The Alaska Tribal Health System is comprised of regional tribal health organizations who collectively are responsible for providing health care to the approximately 150,000 Alaska Native and American Indian people in the state, many of whom live in small remote communities located off the road system. In addition to geographic challenges to health care delivery in Alaska, there are also structural issues with staffing shortages, high turnover of providers, and chronic underfunding of health services for this population [7].

CRC screening adherence is strongly influenced by healthcare provider recommendations [8,9]. Colonoscopy has been the main screening method recommended in the Alaska Tribal Health System, with limited use of at-home stool testing due to sub-optimal test performance of the older guaiac-based fecal occult blood tests in this population [10,11,12]. However, colonoscopy is not widely accessible, especially in rural/remote areas of Alaska. There is a need for additional screening tests to help expand the options available. In 2014, the multi-target stool DNA (mt-sDNA) test (Cologuard^®^, Exact Sciences, Madison, WI, USA), became commercially available for at-home CRC screening for average-risk patients without a personal or family history of CRC.

Previously, we surveyed patients and providers in three rural/remote Alaska Native Tribal Health regions about perceived barriers to colonoscopy and mt-sDNA, which was not yet available in the Alaska Tribal Health System [13]. The top barriers to colonoscopy reported by patients were travel (44%) and bowel preparation (40%), while providers felt that fear of pain (92%) and invasiveness (87%) were the top barriers for their patients. Patients’ top barriers to using mt-sDNA were the belief that colonoscopy is better (56%) and difficulty performing the test (40%), while providers cited collecting stool samples (67%) and having a stool sample in the patient’s home (63%) as top barriers. Overall, the survey showed that patients and providers would consider using mt-sDNA. However, the survey was comprised of primarily close-ended questions so did not allow for in-depth participant responses. The survey also did not ask about facilitators to screening or potential programmatic work that could be conducted to improve screening at the health organization level.

The current study complements our previous work on patient-level barriers by examining the provider- and system-level factors that support or hinder Alaska Native CRC screening uptake from the perspective of healthcare providers (physicians, advanced practice providers, and Community Health Aides/Practitioners) and administrators at the Yukon Kuskokwim Health Corporation (YKHC). This regional tribal health organization serves Alaska Native and non-Native people living in southwest Alaska, in an area covering 75,000 square miles, roughly the size of Oregon with 58 rural communities (~25–1600 residents each) and no road access. YKHC operates 41 village clinics, 5 sub-regional clinics, and a regional hospital located in Bethel, Alaska. The village clinics are primarily staffed by Community Health Aides under the supervision of advanced practice providers and physicians at the hospital and the sub-regional clinics [14]. Intermittent colonoscopy clinics are conducted at YKHC in Bethel or provided via referral at the Alaska Native Medical Center in Anchorage. The YKHC clinic uses endoscopy staff who fly to Bethel, and patients typically travel from the villages by plane, boat, or snow machine. Screening colonoscopies for Alaska Native men and women ages 40–75 years are provided with no co-payment required from the patient, but travel expenses and lodging for the patient and their required medical escort are not covered by the health system.

The study design was guided by the Reach Effectiveness Adoption Implementation Maintenance (RE-AIM) framework [15,16,17,18]. The overall study goal is to identify feasible and culturally appropriate solutions for promoting CRC screening uptake and to inform community and healthcare system efforts to reduce CRC among the Alaska Native population.

## 2. Materials and Methods

This study was approved by the Alaska Area Institutional Review Board and the tribal research review committees at the Alaska Native Tribal Health Consortium (ANTHC) and YKHC. The full intervention study is registered with ClinicalTrials.gov (NCT04336397).

### 2.1. Recruitment and Eligibility

Key informant interviews with Alaska Tribal Health System providers were conducted from 1 February 2021 to 30 November 2021. Eligible participants were healthcare providers involved in CRC screening promotion and/or delivery. A snowball sampling technique [19] was used to reach a target of 25–30 interviews, until saturation was achieved [20]. Recruitment was conducted through e-mail listservs, provider meetings, Community Health Aide training sessions, and word of mouth. Potential participants contacted the study interviewer to schedule a phone interview and were sent a link to a secure HIPAA-compliant Research Electronic Data Capture (REDCap) database [21] where they gave informed consent and provided demographic information (sex, provider type, and community of residence). After the interview, participants received a USD 25 gift card in appreciation for their time.

### 2.2. Materials

The key informant interview materials, including consent form, questionnaire, and moderator and question guides, were refined with input from the ANTHC Research Consultation Committee (composed of 21 Alaska Native/American Indian staff) and regional tribal health partners. Mayo Clinic clinical co-investigators with experience in qualitative study design also contributed to the guide. The interview guide consisted of two sets of open-ended questions and probes, one focused on colonoscopy (the primary CRC screening test in the region) and the other on mt-sDNA (not used for general screening within the health system at the time of the interviews).

### 2.3. Data Collection

Key informants were queried about barriers and facilitators for colonoscopy and mt-sDNA at the provider- and system-level. Key informants also provided suggestions for improving screening organizationally using each test and discussed challenges to screening program improvement. Interviews were recorded using the Rev Call Recorder app (www.rev.com) and transcribed verbatim with Temi software (version 2.4) (www.temi.com) [22]. The audio files were reviewed and transcripts corrected for errors. The study used an inductive approach, allowing codes to emerge from the data, and then consolidating related codes into broader themes [23,24]. The data were analyzed thematically using an inductive qualitative methodology and Dedoose software (version 9.0.107) [25,26]. The codebook was created by the lead interviewer and reviewed by a second staff member. An inter-rater reliability test was conducted on a 10% sub-sample with the two coders, resulting in a Cohen’s kappa statistic of 0.93, indicating acceptable reliability and consistency in applying codes to excerpts [27].

## 3. Results

A total of 28 in-depth key informant provider and healthcare staff interviews were completed. These included physicians (18%), advanced practice providers (39%), Community Health Aide/Practitioners (18%), case managers (11%), and administrative staff (14%). The largest group of providers (57%) worked at the regional hospital in Bethel, AK, while 29% were employed at sub-regional clinics and 14% worked in remote village clinics. Informant ages ranged from 28 to 62 years (Median age = 49 years) and the majority of key informants identified as female (79%). Interview length ranged from 12 to 79 min (average = 24 min). Table 1 presents themes and sub-themes for provider- and system-level barriers to using colonoscopy and mt-sDNA for CRC screening.

### 3.1. Provider Level Barriers to Colonoscopy

#### 3.1.1. Time Constraints and Competing Priorities

Respondents identified several provider-level obstacles including a focus on acute care and limited time during patient visits which makes it difficult for them to carry out preventive work such as checking on CRC screening status. Providers noted that the primary focus during most appointments is on acute care, particularly when clinics are short-staffed: *“Right now, we are understaffed. We have a village with about 1600 people. We only have two providers on and we had two health aides. Today is actually one of the health aide’s last days. We’re going to be down one more provider… I feel like as a provider, I am in survival mode all the time”.*

#### 3.1.2. Insufficient Patient Navigation

Providers noted that the lack of patient navigation and case management support has led to reduced completion rates for colonoscopy procedures. One provider in a village setting commented, “*That is a barrier, not having that person or lack of coordination or monies or funds for the health corporation to have that type of people to reach out”.* Several providers also noted the absence of a program that had previously identified and reached out to patients with a family history of CRC. Providers expressed concern regarding the lack of coordination, funds, and personnel to address this issue.

#### 3.1.3. Inadequate Education and Outreach for CRC Screening

Respondents identified outreach and education as key strategies for improving CRC screening in both clinical and community settings. Providers felt that patients with a family history of CRC are generally aware of the need for screening, whereas those without such personal experience lack this awareness. A village-based advanced practice provider stated, *“People that don’t seem to have some kind of personal connection with it, I don’t know if they truly understand that prevalence out here—how high it is and how important it is to find things early”.*

#### 3.1.4. High Provider Turnover Hinders Continuity of Care

In addition to being short-staffed, healthcare providers reported a high rate of turnover at the organization. This results in inadequate knowledge of their patients’ medical history, including whether they require screening or if they have had one in the past: *“You just see anybody and you don’t know that person for continuity of care”.* Additionally, some respondents noted that while a few providers have remained in the region for many years, others are more transitory, especially if they come earlier in their career under the Indian Health Service loan forgiveness program, and the organization relies on locum and intermittent providers to help fill in the gaps. These issues present a significant challenge to the continuity of care and follow-up for necessary screening procedures.

### 3.2. System Level Barriers to Colonoscopy

#### 3.2.1. Absence of Automated Colonoscopy Order Reminder in the Electronic Health Record (EHR)

Challenges in care continuity are further compounded when providers require additional time to locate information about past screenings in the medical chart. Several respondents highlighted that the absence of an automated reminder “flag” in the patient medical record makes it easy to overlook ordering colonoscopies and discussing screening with patients: *“When someone [is age-eligible], nothing is triggered. Nothing. Like, I mean like a light doesn’t go off on their chart”*. This lack of automated reminders creates a gap in the delivery of preventive care services and highlights the need for more effective EHR prompts to encourage providers to recommend screening according to guidelines.

#### 3.2.2. Challenges Retrieving Colonoscopy Results from the EHR

Difficulty accessing past colonoscopy results further complicates the screening process, particularly when the colonoscopy was conducted at the Alaska Native Medical Center in Anchorage, Alaska, which uses a different EHR system from the regional hospital. Primary care providers also face time constraints and may overlook screening tests or pass on referrals without full assessment. As one provider put it, *“I’m not going to spend an hour of my time looking for that colonoscopy. I figure those people at the colonoscopy suite, they can figure out if this person had one [previously]”.* Providers also noted that they do not receive feedback such as overall referral rates for themselves or whether individual patients ever received the colonoscopy for which they were referred.

#### 3.2.3. Lack of Coverage for Travel Expenses for Screening Colonoscopy

Although CRC screening (stool testing or colonoscopy) is covered by the tribal health system, according to the healthcare providers surveyed, the lack of Medicaid or YKHC coverage for the cost of transportation for colonoscopy poses a significant system-level barrier. A staff member described the impact of this barrier: *“When you are living in Hooper Bay and you’ve got an $800 flight [to get to the colonoscopy clinic] that is a pretty significant investment to go into Anchorage or to go to Bethel. So, if they can put it off, they’re going to put that off”.* Since Alaska Medicaid covers the travel expenses associated with a diagnostic colonoscopy for patients enrolled in Medicaid, providers will sometimes recommend an at-home stool test so that if it is abnormal the patient’s travel and procedure are covered for the follow-up diagnostic colonoscopy. However, providers felt that adherence to completing stool tests is low. The lack of coverage for travel expenses also complicates the scheduling process, as providers feel obligated to make referrals despite knowing that patients may not be able to afford the travel. This results in high no-show rates, which take up appointment slots and impede colonoscopy procedures for other patients. To address this challenge, some providers and schedulers attempt to combine colonoscopy travel with other procedures where travel expenses are covered.

#### 3.2.4. Clinic Cancellations Due to Inclement Weather and Lack of Local Staff

The colonoscopy clinic in the hub community faces unique logistical challenges due to its remote location and reliance on itinerant endoscopy providers. Surgeons and nurses have to fly to the hub community to provide colonoscopies, but bad weather in the winter can often delay or cancel their flights, resulting in clinic cancellations: *“That’s a whole day of scheduled patients that are prepped…. so every time you get people in for a screening colonoscopy you’re kind of hit with all of these logistical factors too that you wouldn’t normally see on the road system or in a regular practice”.*

#### 3.2.5. Impact of the COVID-19 Pandemic on Colonoscopy Wait Times

The COVID-19 pandemic, which caused travel closures between communities and paused elective procedures, had a profound impact on colonoscopy wait times, leading to an unprecedented backlog of referrals. Key informants noted that there are hundreds of patients on the diagnostic colonoscopy schedule list, and several thousand more on the screening list. A staff member who works with the colonoscopy clinic explained that a high no-show rate exacerbates the backlog of appointments: *“With the backlog, we schedule all these patients and our no-show rate is quite high, sometimes greater than 50%. Then those people just go right back on the list and the no-shows contribute to make the backlog even longer because now those people have to be rescheduled again and put back on the list”*. Another clinic staff member said, *“Like you might get four colonoscopies done out of 12 that are scheduled. And they should be doing up to 20 a day. The providers who are hired to do colonoscopies—they want to do colonoscopies and they are sitting on their hands. That’s because of all the things that are outside of the medical aspects of it”.* The backlog of patients needing diagnostic procedures makes it difficult for providers to make screening referrals, especially for average risk patients: *“They always tried to put the most urgent at the top of the list. But that means that a lot of people weren’t getting routine scopes then because there was never a spot, unless you just got lucky at the right time and there was a cancellation, and you were nearby or something”.*

### 3.3. Potential Barriers to Screening with mt-sDNA

Provider key informants were asked about mt-sDNA as a potential new alternative to colonoscopy. All providers interviewed said they would recommend mt-sDNA and saw potential for it as a screening option in the region. A village-based Community Health Aide stated, *“Yeah, I think that would work because you know, they’d be in their own home and they would be the one handling their stool. So I think they’d feel more comfortable doing that than going through the clinic”.*

#### 3.3.1. Potential Delay in Receiving Test Kit within the Required Time

Many providers expressed concerns about the timely arrival of mt-sDNA samples to the laboratory via mail flights. During the study period, the time from collection to laboratory receipt needed to occur within 72 h, although the current version of the test is viable for up to 96 h from sample collection. Providers pointed out that weather conditions in the region can often cause significant delays in mail delivery. A village-based Community Health Aide stated: *“I mean—where I live we only have three flights per day, Monday, Wednesday, Friday and it all depends on weather if the mail goes out or not. Sometimes we won’t have a plane for a whole week because of weather”.*

#### 3.3.2. Complex Instructions for Completing mt-sDNA

Many providers expressed concern that the instructions for the test may be overly complex and contain numerous steps, posing a challenge for patients with limited literacy or for those whose first language is not English. One physician stated: *“I have trouble getting [patients] to do the stool blood test, the iFOB, where you just have to puncture your bowel movement five times and stick that in the tube. So having a bowel movement in that container and scraping stuff out, putting in a new tube, putting stabilizer in and then putting it in the packaging, it’s going to be beyond most people”.*

#### 3.3.3. Lack of Adherence to Use of Other Stool Tests

The majority of healthcare providers expressed concerns regarding patient adherence using mt-sDNA and compared it to the immunochemical fecal occult blood (FIT) stool test. Providers stated that they often send patients home with the FIT kit to collect a stool sample, but the patients frequently fail to return the test to the clinic for processing. A physician noted this issue, stating: *“They just don’t end up doing it. We will order it and we will give it to them and they just don’t turn it in. So I don’t know”.* Nonetheless, a few providers mentioned that they had observed successful FIT adherence among their patients.

#### 3.3.4. Electronic Health Record Challenges

Providers noted that the same difficulties with accessing past screening results in the EHR would make it challenging to recommend mt-sDNA to patients. Additionally, mt-sDNA is only recommended for use by average-risk patients without a family or personal history of CRC, which is not well documented in the medical record. Providers said it would take more time to find that information which could be a barrier to use. *“Then, ideally if there were a way for the EMR to be able to identify and provide a trigger for providers and nurses to see who is due for a screening without having to really search and dig for it. That would be great because that would eliminate providers having to search for nurses, having to go in and see when was the last colonoscopy, when was the last, FIT test or whatever was done. So just having some type of physical and, or not physical, —a, visible prompt would be helpful”.*

### 3.4. Respondent Suggestions for Improving CRC Screening

In addition to barriers, provider respondents were asked about facilitators to screening using colonoscopy and mt-sDNA and suggestions on how to improve the screening process. Table 2 presents themes and sub-themes for facilitators to using colonoscopy and mt-sDNA for CRC screening.

### 3.5. Facilitators for Screening with Colonoscopy

#### 3.5.1. Community Outreach

Respondents noted that community members who have undergone CRC screening, including tribal council members and local clinic staff, are trusted messengers for educating the community about the importance of CRC screening. A staff member at a village clinic emphasized the effectiveness of having familiar faces share their screening experiences: *“I think if you have faces that people know who have gone through it tell their stories, it is probably the best way… I think just getting the word out there that people are having [colonoscopies] and it’s not that big a deal and that we should have them for our health is the first thing”.*

To effectively promote CRC screening, respondents suggested focusing on teaching about CRC risk levels, who should be screened, and describing the colonoscopy procedure and preparation. They recommended disseminating the message through various channels, such as the clinic website, social media, the VHF radio or TV, posters, and pamphlets that could be displayed in the clinic waiting room. Some suggested innovative methods for community outreach such as painting dumpsters or murals in the community with CRC screening messages. Respondents emphasized the need for messaging in both Yupik (the Alaska Native language spoken in the region) and English and suggested holding education sessions for the community on CRC, including demonstrations of the equipment used during colonoscopies.

Respondents proposed various strategies for clinics to encourage screenings among the target population, such as scheduling more wellness visits and having providers inquire about screening during each acute care visit. An advanced practice provider explained, *“So, if they only go to the village clinics for acute care and they decline wellness visits because they feel fine, they don’t really get that information unless somebody has time to fit it in on an acute care visit”.*

#### 3.5.2. Electronic Health Record Tools

To facilitate the screening process, respondents suggested using tools within the EHR, such as automatic age-appropriate referrals or screening flags, to help providers track screening-eligible patients. Endoscopists have also been ensuring more systematic follow-up care by putting in the order for the next procedure once the colonoscopy results are completed so that there isn’t a need to hunt through the medical record to see when a patient is next due for screening or surveillance.

#### 3.5.3. Cultural Competency and Patient Navigation

Respondents suggested that an Alaska Native staff member should be the primary contact for recruiting and assisting patients with the screening process. Additionally, providers recommended greater involvement of Community Health Aides who work in village clinics to promote screening and make referrals. Respondents suggested that this could be accompanied by schedulers and patient navigators to work with patients on a personal level, as well as reintroducing an outreach program for those with a family history of CRC. A village provider stated, *“Giving information to a mass group of people is wonderful. It educates the people, but I know that people would like to be personally spoken to and given that information [for colonoscopy preparation], when they talk about when to eat, what not to eat, when not to eat- the berries, the seeds, the colors of the jello, all those things”.*

#### 3.5.4. Clinic and System Improvements

Suggestions to improve colonoscopy screening adherence centered on addressing the screening backlog by hiring or contracting more staff and increasing the number of days that colonoscopies are offered. Additionally, they suggested focusing on the bottlenecks in the system which hinder patients from being screened. Respondents recommended involving all relevant parties from the Community Health Aide at the village clinic to the Medicaid authorization staff to the colonoscopy clinic staff to: *“to make sure they understand one another and to put all the pieces of the puzzle together so that it actually flows”.*

### 3.6. Facilitators for Screening with mt-sDNA

#### 3.6.1. Cultural Competency and Patient Navigation

Respondents recommended developing an outreach strategy in consultation with Alaska Native elders on how to promote mt-sDNA use effectively. This would include selecting appropriate terminology for stool and discussing the concept of DNA in a culturally appropriate manner. Respondents suggested providing education in both Yupik and English and conducting outreach at community events such as dog mushing races and festivals. Visual aids like birthday cards and CDs could also be utilized, and local teachers could be trained to teach about the test in science classes. A village physician stated, *“It’s persistence and constant education—getting at people over and over again. I believe there are CDs out there that are in Yupik we need to make available to people. Just all the information and making them aware”*. A Community Health Aide suggested, *“People watch TV all the time…maybe ask the local TV channel if they could air something short—something on the importance of screening”*.

Moreover, respondents proposed that test instructions should be more straightforward, with fewer steps, written in non-medical language, and available in both Yupik and English. Patients should be consulted on how to word the instructions. Respondents also felt that gloves should be included in the kit, and people should be incentivized for completing the kit. Some providers recommended face-to-face education about the kit and suggested demonstrating the process through a mock demonstration or cartoon video.

To make the kit accessible to patients, several options were proposed, such as distributing it in the community through spaces like grocery stores or the health clinic, or home delivery with clinical staff available to answer questions. Additionally, patient navigators would be helpful to assist individuals in completing the test successfully. Local champions were also recommended to help increase community member buy-in and interest in the screening test.

#### 3.6.2. Clinic and System Improvements

Regarding clinic activities, respondents recommended clinical staff training on the new test to be able to instruct patients on it effectively. Respondents believed that clinic staff’s active participation would be necessary for successful completion of the test, including potentially having patients do the stool collection at the clinic and mailing in the completed kit for the patient. One provider stated: “*I think being able to do this and having the option of maybe doing it at the clinic where somebody can help and get it set up for shipment would be—you [would] have more success. I think than having somebody try to do this on their own”*. However, other providers believed that patients being able to mail the sample would be better: “*I think mailing, it would be much less embarrassing than bringing back their stool to the clinics”.*

Respondents also suggested incorporating more preventive care into the regional healthcare system, such as elder clinics or scheduled elder wellness visits: *“One thing that I’ve wondered whether it could improve our compliance on more things, not just the colorectal screenings would be maybe to open up a slot specifically for basic healthcare maintenance—elder health exams […] The records could be reviewed in advance and we already know what they need when they walk in the door”*.

Suggestions for implementing the new screening tool on a system-wide level included designating sub-regional clinics as responsible for the process for communities in their service area. One provider proposed a program similar to COVID vaccination: “*I would just send people out just like you do with vaccinations. That we did with pandemic response. If you want to do screening, just go and hit one type of screening at a time. And the fortunate thing with colorectal screening with the stool DNA test—with the hemoccult cards. You can do that. You can collect all the samples there. You can’t do that with colonoscopy”.* Finally, a respondent suggested that sharing statistics on how many kits have been completed and the results would give people confidence in the effectiveness of the screening tool.

## 4. Discussion

This study confirmed the commitment of providers serving Alaska Native people to address CRC disparities. Barriers are well known and significantly, key informants provided creative and practical suggestions for improving services for prevention and early detection. Provider key informants identified barriers at both the provider- and system-level for colonoscopy and potential barriers for mt-sDNA. While some barriers overlapped between the two tests, each screening test has its unique barriers that need to be addressed separately. For colonoscopy, the challenges include high travel costs, weather disruptions leading to clinic cancellation, and long waitlists for patients, exacerbated by high no-show rates. For mt-sDNA, the concern is that the test may not be completed, mailed, and received by the lab within 96 h. It is worth noting that mt-sDNA would be added to an existing CRC screening program that uses an EHR to record referrals and test results, but providers reported difficulty accessing screening history and follow-up recommendations in the EHR. This suggests that overall screening rates could be improved regardless of test option if the EHR could alert providers to patients’ screening needs and results, as well as any necessary follow-up.

Our findings that high staff turnover and an acute care focus, coupled with a lack of time during appointments, hinders screening promotion efforts are concordant with a national study of primary care clinicians and gastroenterologists [28], although that study also noted providers not proactively recommending screening and not having sufficient education about the types of screening options available as barriers. To overcome barriers, respondents suggested training front-line health staff at village clinics in CRC prevention and involving them more in education and screening recruitment. Additionally, case management/patient navigation was recommended for both types of screening to assist patients and facilitate screening adherence, in line with strategies recommended among other tribal and non-tribal populations [28,29,30,31]. Having dedicated staff focused on CRC screening would help to decrease the patient no-show rate. Increasing screening completions would help provider and staff job satisfaction and potentially decrease staff turn-over.

Many of the screening facilitators suggested apply equally to both tests. The respondents suggested that community outreach and grassroots efforts be implemented to educate people about the risk of CRC, the importance of screening, and the specifics of each test. This same approach was suggested in a study on cancer prevention in North Dakota tribal reservation communities which recommended using community role models or spokespersons for cancer prevention and leveraging storytelling, traditional knowledge, and ceremony to disseminate the prevention message and engage with the community [32]. Providers in our study were also asked about language as a screening barrier since many Alaska Native elders in the region still speak their Native language at home. Providers gave mixed responses on whether this is a barrier, which should be explored further with Alaska Native community members.

Some study limitations should be noted. Although a number of barriers, including structural barriers, were brought up by providers interviewed, there are likely others that affect CRC screening. The overall number of respondents was small, primarily women, and came from only one tribal health system, so it is not possible to generalize these findings to other providers and health systems. The study used a snowball sampling frame, which resulted in interviewing about 12% of the total providers within the health system. Therefore, other providers may have different sociodemographic or other characteristics, and their perceived provider- and system-level barriers and facilitators may also differ. Additionally, the racial/ethnic status of participants was not assessed, so it is not possible to determine differences in perceived barriers and facilitators by tribal affiliation. There was also a modest number of interviews with providers in village clinics (four), who may encounter different challenges compared to respondents at the larger sub-regional clinics and the regional hospital. However, it is worth noting that this study is the first of its kind to explore in-depth provider perspectives on using colonoscopy and mt-sDNA for CRC screening among Alaska Native people, and identified a number of factors that could be proactively addressed to help increase CRC screening in this population.

## 5. Conclusions

In-depth interviews with tribal health system staff showed that incorporating mt-sDNA as a screening option for CRC could be advantageous in addressing system-level barriers such as lengthy appointment waitlists and expensive travel costs for screening colonoscopy. However, other system-level barriers would still require attention and improvement to facilitate screening using mt-sDNA. Further research is needed to evaluate individual-level patient barriers and facilitators for both colonoscopy and mt-sDNA, as well as the effectiveness of integrating both tests into a geographically dispersed tribal health system to reduce cancer disparities and build equity for cancer prevention among Alaska Native people.

## Figures and Tables

**Table 1:** Themes and sub-themes of provider and system barriers to CRC screening with colonoscopy or multi-target stool DNA.

Theme	Sub-Theme
Provider barriers to colonoscopy	
Time	Clinical exam time constraints Insufficient time for patient navigation
Competing priorities	Busy managing other patient health issues Emphasis on acute care versus prevention Limited involvement of frontline workers in promoting CRC screening Inadequate education and outreach for CRC screening
Staffing	Short-staffed clinics High provider turnover Reliance on locum and temporary providers Discontinuity of care
System barriers to colonoscopy	
Electronic health record	No automated colonoscopy order reminder Hard to find screening history Hard to find colonoscopy results and next steps Lack of provider assessment and feedback
Cost	Health system doesn’t cover travel for patient or escort Lack of Medicaid coverage for screening colonoscopy travel
Weather and seasonal activities	Clinic cancellations due to inclement weather Travel delays making patients reluctant to get screened Traditional food gathering activities taking precedence
COVID-19 pandemic	Longer colonoscopy wait times Colonoscopy backlogs
Barriers to mt-sDNA	
Limited test viability	Potential delay in laboratory receiving test kit within the required timeframe for analysis
Unfamiliarity with test	Complex instructions for completing mt-sDNA
Experiences with other stool tests	Lack of patient adherence with other stool tests
Electronic health record	No automated screening order reminder Hard to find previous screening results Hard to determine if patient eligible for stool testing

**Table 2:** Themes and sub-themes of facilitators to CRC screening with colonoscopy or multi-target stool DNA.

Theme	Sub-Theme
Facilitators for colonoscopy	
Community outreach	Increase use of trusted community members to spread screening message Use different channels to get the message out (radio, TV, posters) Hold community events to demystify CRC screening Increase wellness visits and prevention reminders during acute care visits
EHR tools	Assess patient screening status and eligibility Automatic referrals EHR screening reminder “flags”
Cultural competency and patient navigation	Have Alaska Native staff as primary contact for screening Increase Community Health Aide involvement in screening Hire dedicated patient navigators to promote screening
Clinic and system improvements	Address screening backlog by hiring more staff Increase the number of days that colonoscopies are provided Review screening process flow to identify bottlenecks in the system
Facilitators for mt-sDNA	
Cultural competency and patient navigation	Consultation with Alaska Native Elders Outreach at community events Test instructions in Alaska Native language Alternate test instruction formats such as in-person demonstrations or short videos Have kits available at clinic or community locations Patient navigators Local champions
Clinic and system improvements	Train providers on use of mt-sDNA Providers facilitating test completion Hold elder clinics and wellness visits Designating facilities that are responsible for promoting the test Community wide campaigns, similar to COVID vaccination efforts Sharing number of tests completed and cancers/precancers found

## Data Availability

Data are not available due to tribal research data restrictions. Researchers interested in these data may contact the corresponding author for more information on the process for requesting access to these tribal data.
